# Supplementary figures and images for: Phosphorylation of the N-terminus of Syntaxin-16 controls interaction with mVps45 and GLUT4 trafficking in adipocytes
Source: PeerJ. 2023 Jul 24;11:e15630. doi: 10.7717/peerj.15630 (PMC10373645; doi:10.7717/peerj.15630)

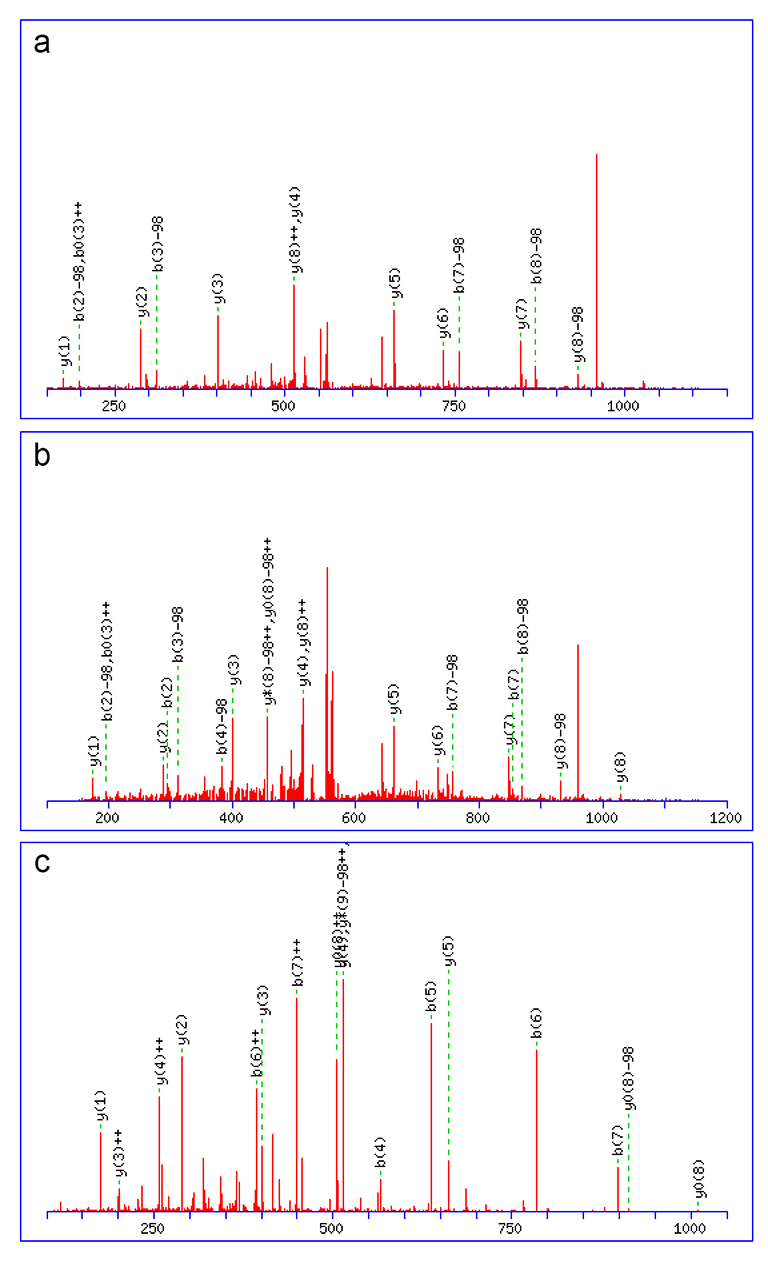

Supplement: Supplemental Information 1 — Original MS spectra of the three species shown in Fig. 1 are presented [file peerj-11-15630-s001.jpg]

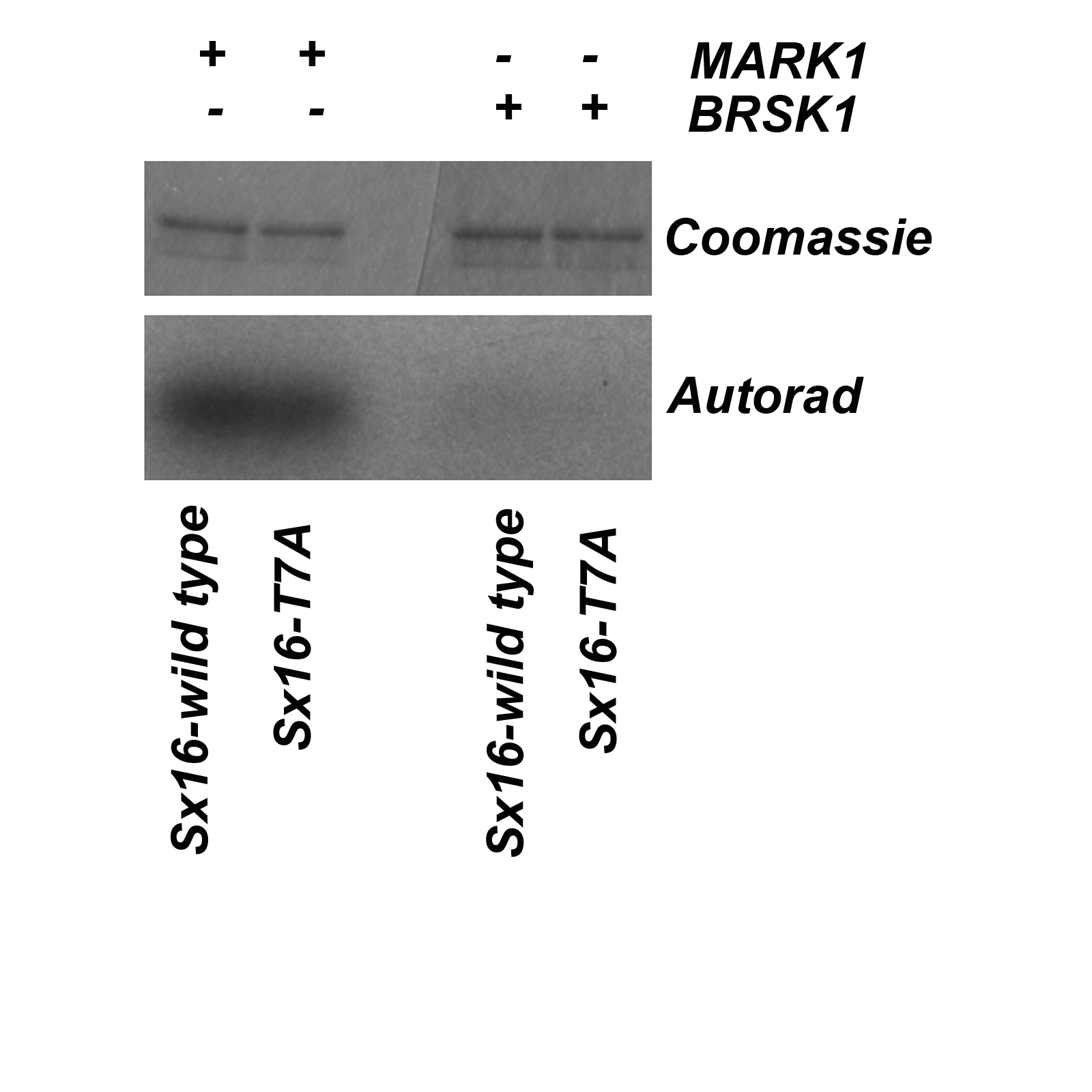

Supplement: Supplemental Information 2 — Shown are data from an in vitro kinase assay using wild-type Sx16 or Sx16-T7A incubated with MARK1 or BRSK1 kinases as described. MARK1 phosphorylatedSx16, but this was not reduced in the T7A mutant and thus suggests this kinase targets a distinct site. BRSK1 was an example of a kinase that did not phosphorylate Sx16. [file peerj-11-15630-s002.jpg]
