# Supplementary material for: Phosphorylation of the N-terminus of Syntaxin-16 controls interaction with mVps45 and GLUT4 trafficking in adipocytes
Source: PeerJ. 2023 Jul 24;11:e15630. doi: 10.7717/peerj.15630 (PMC10373645; doi:10.7717/peerj.15630)
Supplement: Supplemental Information 4 [file peerj-11-15630-s004.docx]

**Figure 2B**

Original blot file:


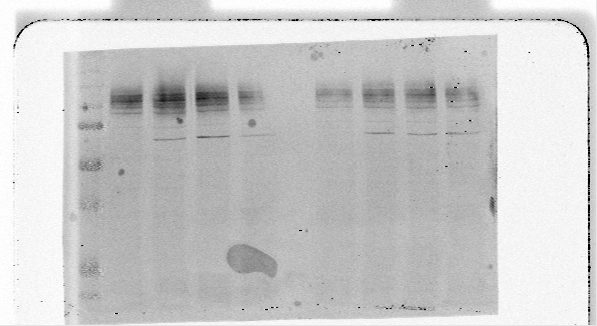


Shown is anti-HA immunoblot, which detects both HA-tagged GLUT4-GFP (upper smear) and HA-mVps45 (band at roughly 68 kDa).

**Figure 3A**

Original autoradiograph:


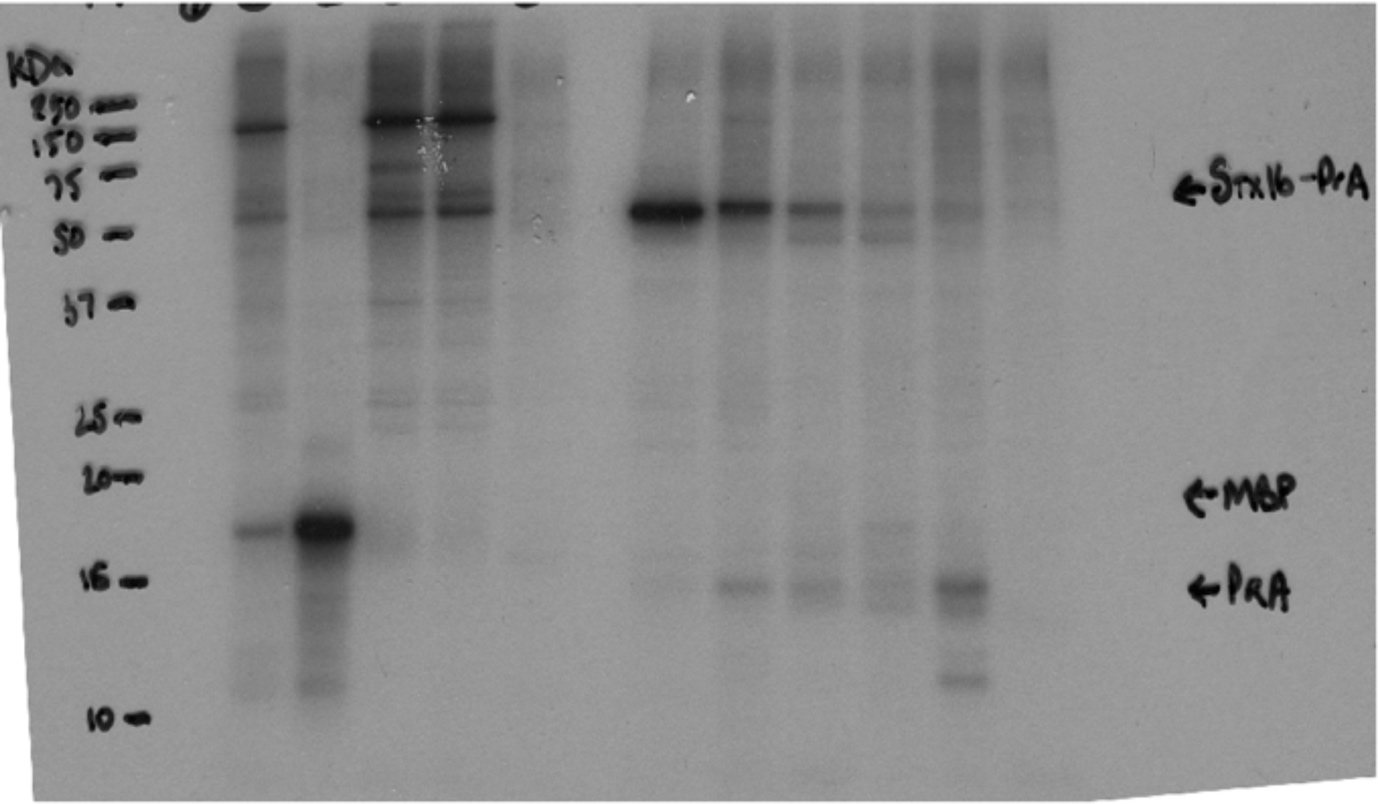


Samples shown in figure are boxed. Other lanes are either controls or experiments using different constructs not used in this study.

**Figure 3B:**

Original blot file:


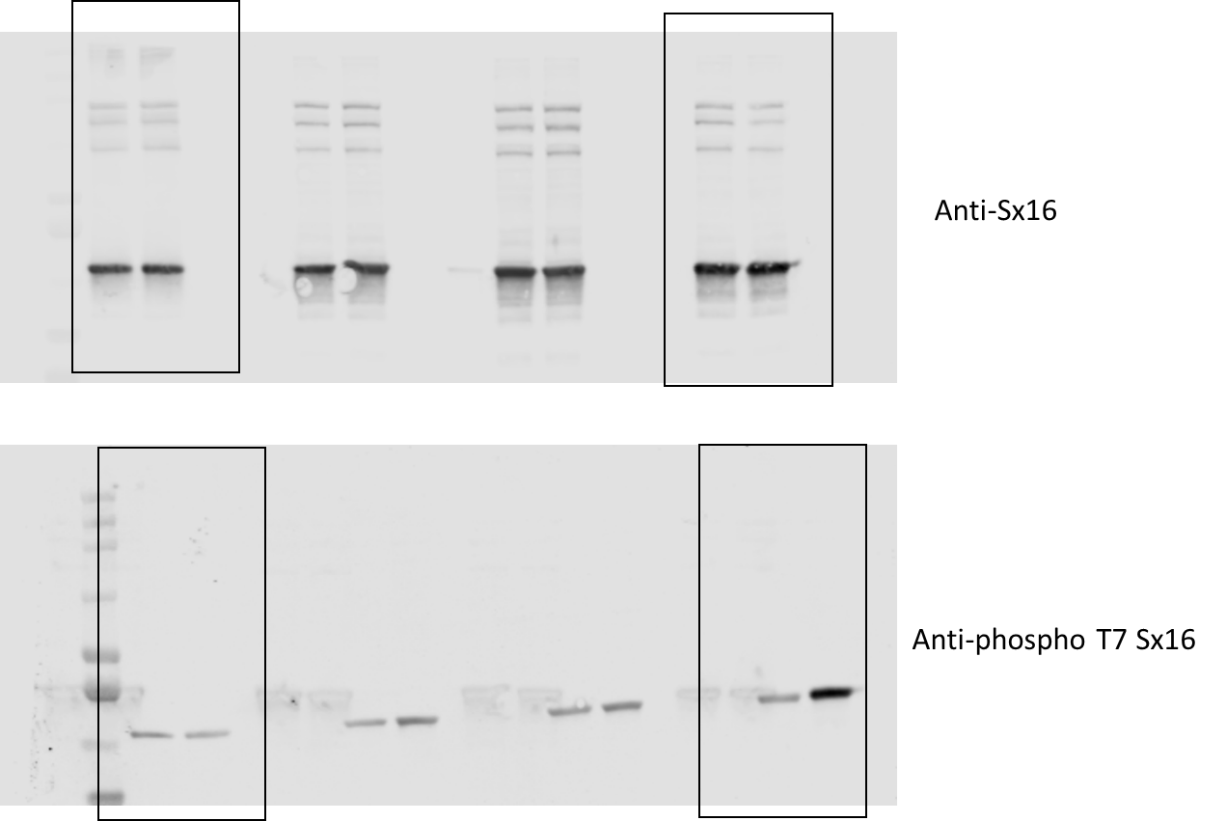


Shown is anti-Sx16 and anti-phospho T7 Sx16 immunoblot. Samples shown in figure are boxed. Other lanes are experiments using different conditions not used in this study.

**Figure 3C:**

Original blot file:

**
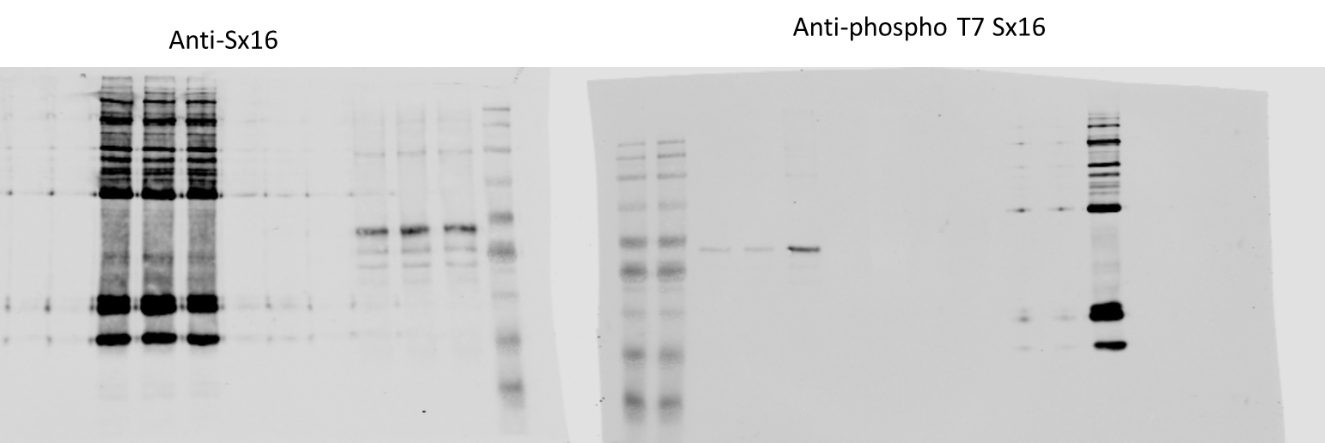
**

Shown is anti-Sx16 and anti-phospho T7 Sx16 immunoblot.

**Figure 4:**

Original stain and blot files:


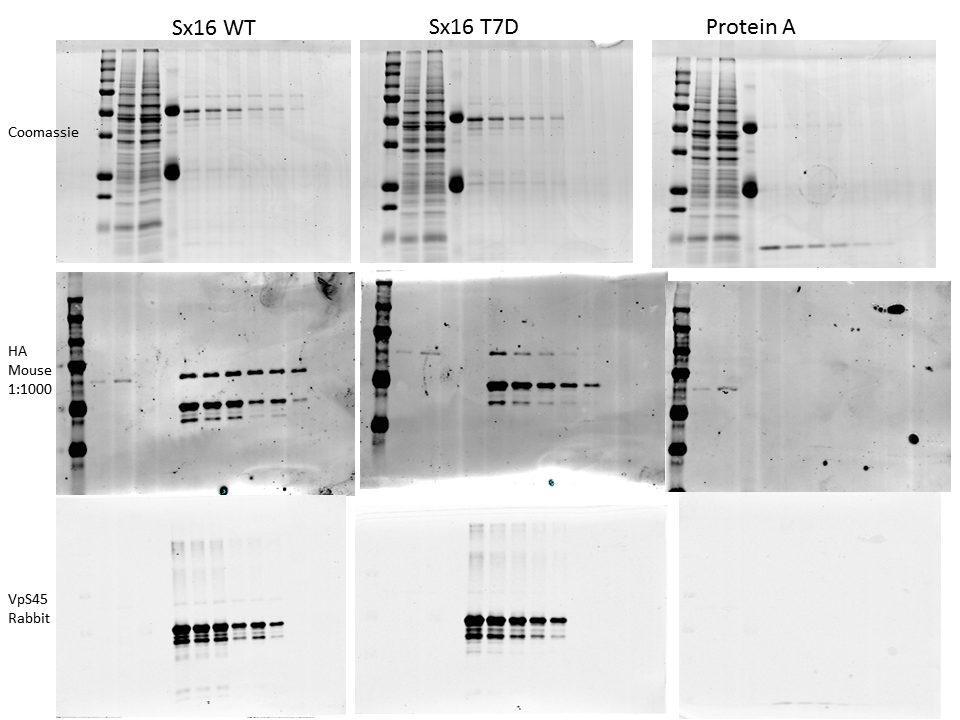


Samples shown in figure are boxed. Images show Coomassie stained gels and anti-HA immunoblots.

**Figure 5**

Original blot files:

Anti-GLUT4


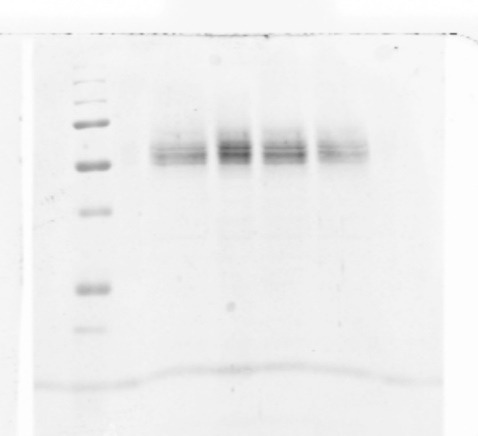


Anti-mVps45


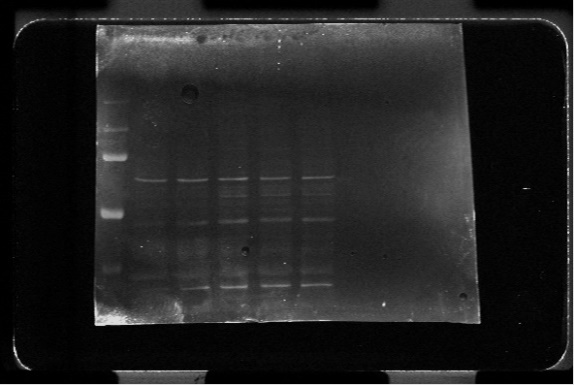


Anti-Sx16


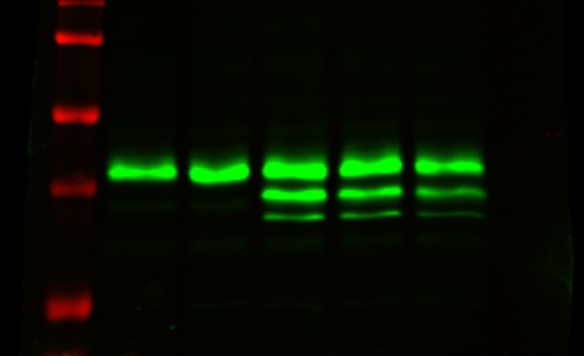



Anti-GAPDH

Shown are anti-Sx16, anti-mVps45, anti-GLUT4 and anti-GAPDH immunoblots. In order, samples are; positive control (present in anti-mVps45,-Sx16 and -GAPDH immunoblots), control cells, WT Sx16,T7A Sx16 and Sx16 T7D. Note for the anti-GAPDH immunoblot there is an empty lane between positive control and experimental samples which was run on a separate gel to those above because of size clashes.

**Figure 6**

Original Coomassie Blue stained gel and autoradiograph:

**
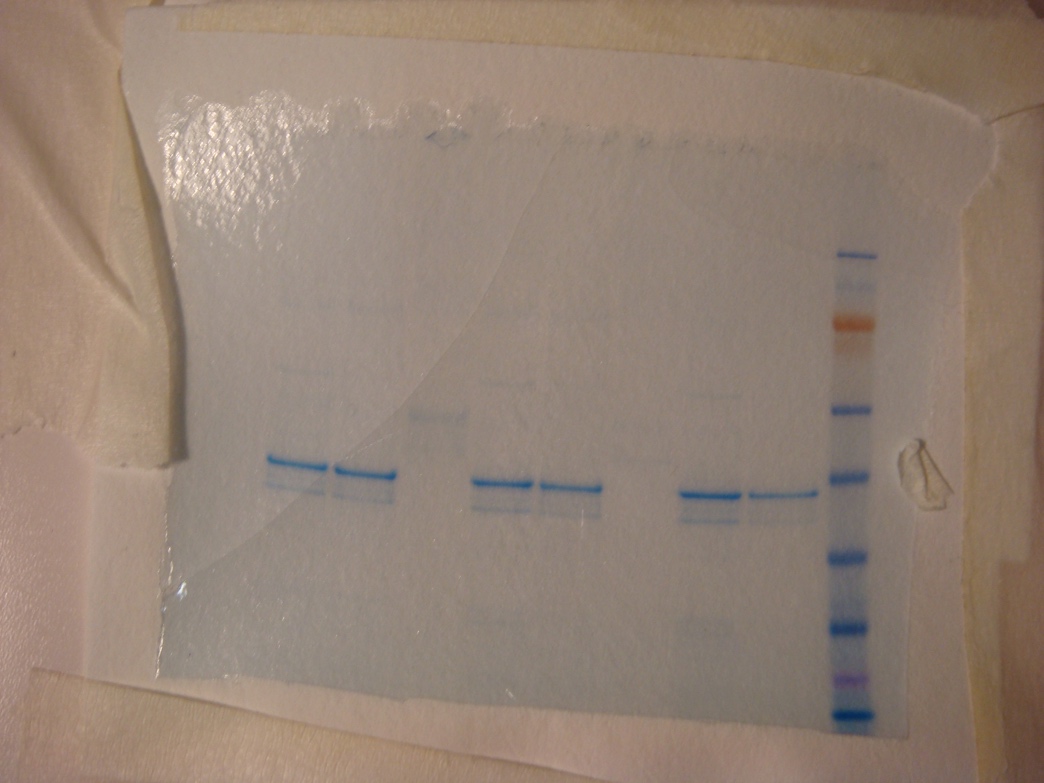
**

Corresponding Autorad.

**
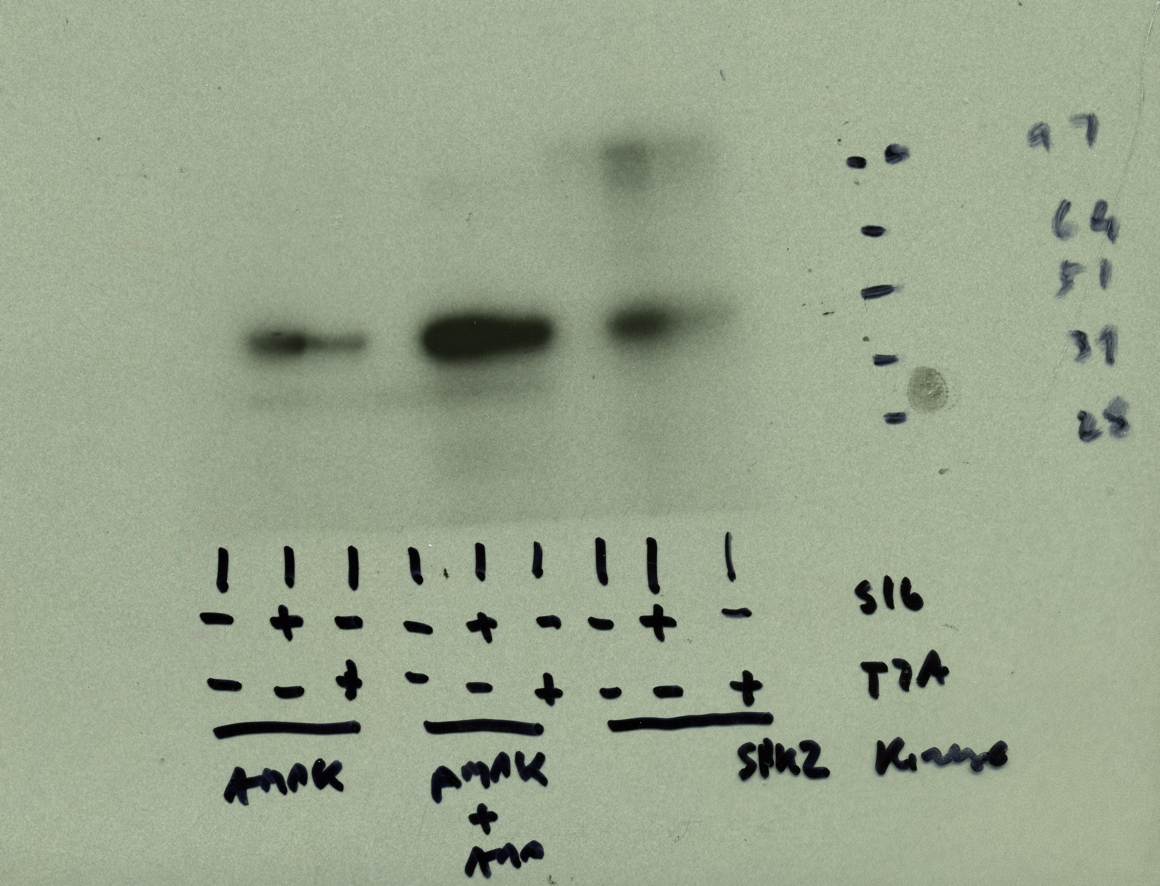
**

Shown is the original Coomassie Blue stained gel and the accompanying autoradiograph.

**Figure 7A:**

Original blot file:


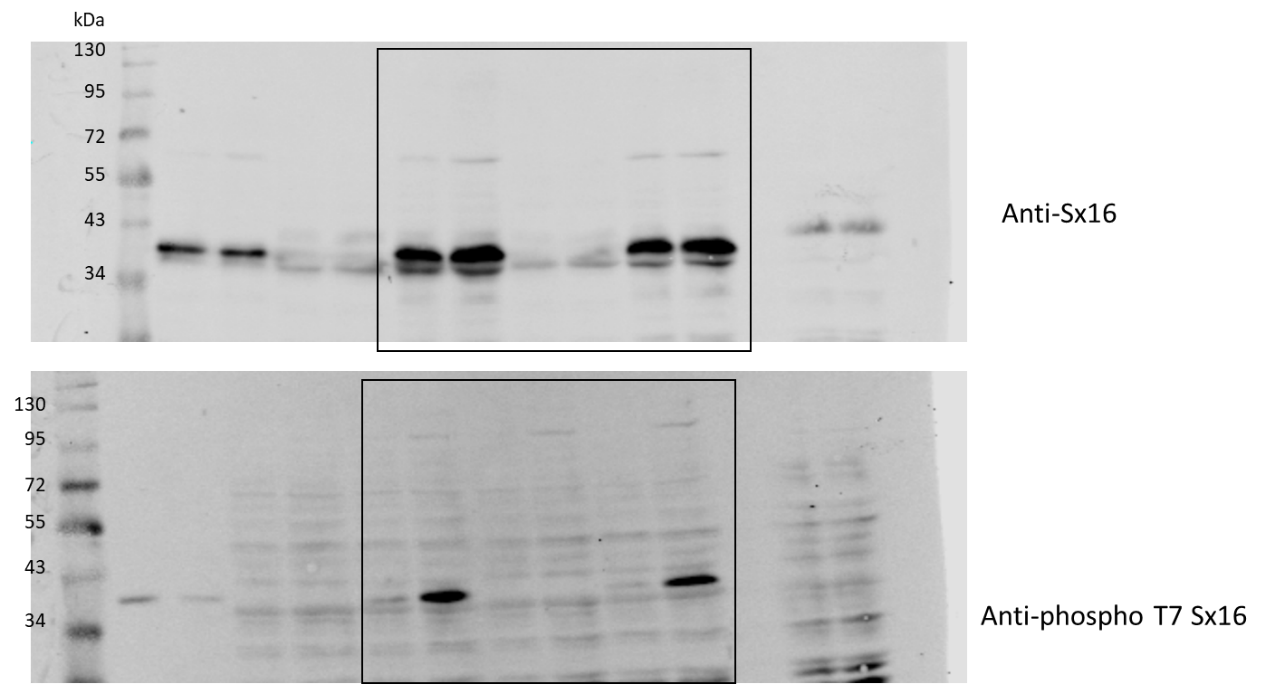


Shown is anti-Sx16 and anti-phospho T7 Sx16 immunoblot. Samples shown in figure are boxed, other lanes are experiments using different conditions not used in this study.

**Figure 7B:**

Original blot file:


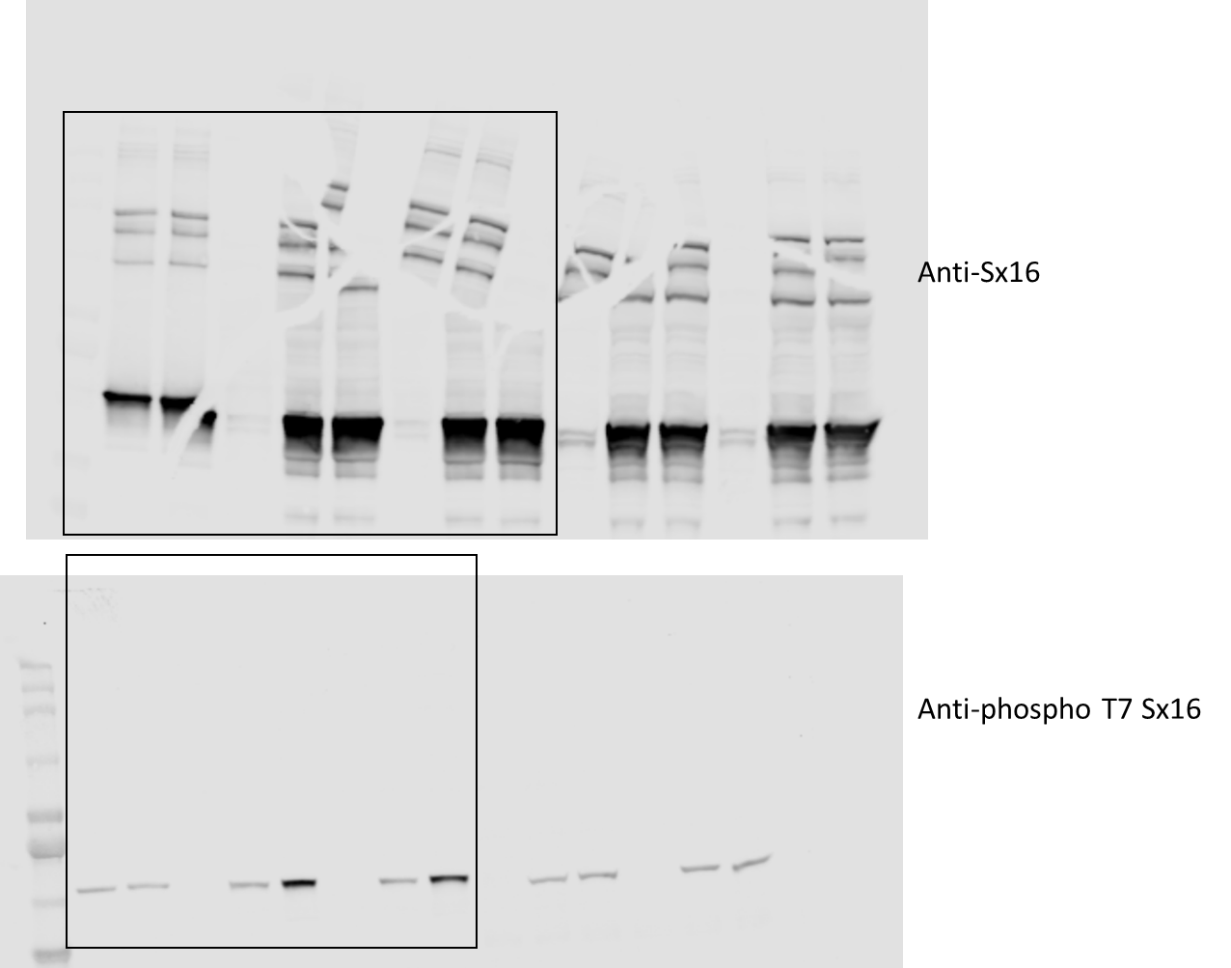


Shown is anti-Sx16 and anti-phospho T7 Sx16 immunoblot. Samples shown in figure are boxed, other lanes are experiments using different conditions not used in this study.

**Figure 7C:**

Original blot file:


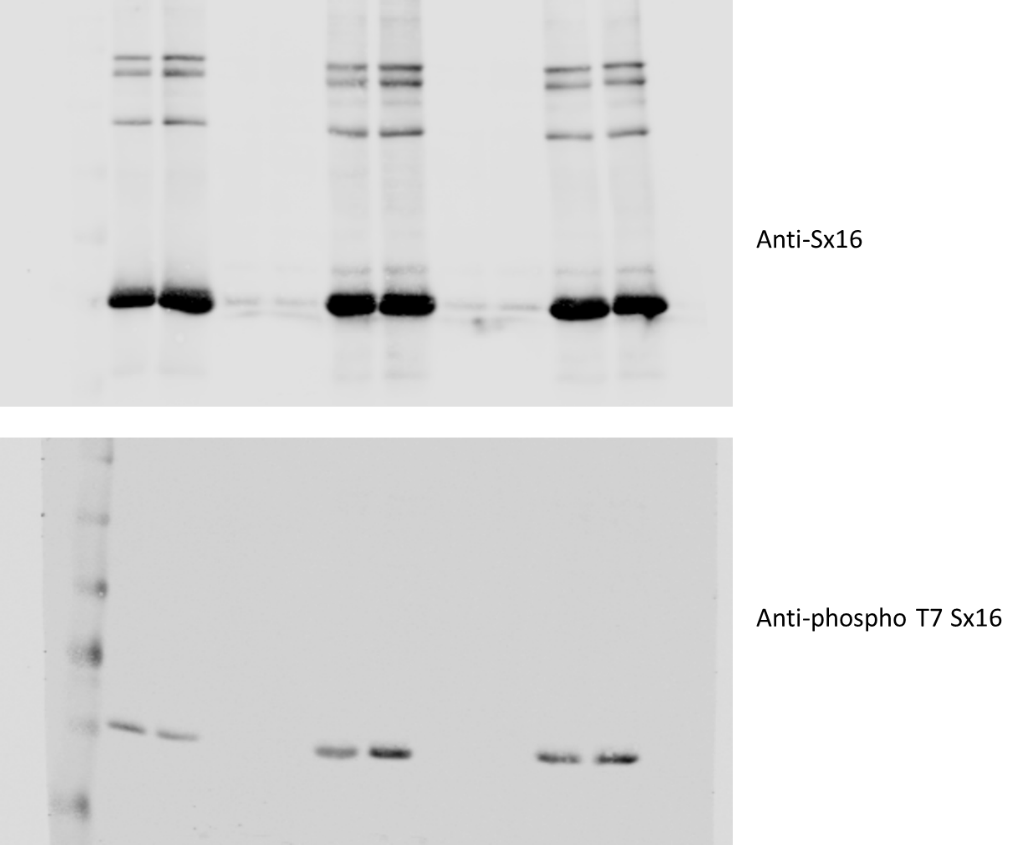


Shown is anti-Sx16 and anti-phospho T7 Sx16 immunoblot.
